# Two-Way Characterization of Beekeepers’ Honey According to Botanical Origin on the Basis of Mineral Content Analysis Using ICP-OES Implemented with Multiple Chemometric Tools

**DOI:** 10.3390/foods8060210

**Published:** 2019-06-14

**Authors:** Artemis Panormitis Louppis, Ioannis Konstantinos Karabagias, Chara Papastephanou, Anastasia Badeka

**Affiliations:** 1 CP. Foodlab Ltd., Polifonti 25, Strovolos, 2047 Nicosia, Cyprus; artemislouppis@gmail.com (A.P.L.) foodlab@cytanet.com.cy (C.P.); 2 Laboratory of Food Chemistry, Department of Chemistry University of Ioannina, 45110 Ioannina, Greece; abadeka@uoi.gr

**Keywords:** characterization, beekeepers’ honey, minerals, inductively coupled plasma optical emission spectrometry (ICP-OES), chemometrics

## Abstract

Asfaka, fir, flower, forest flowers and orange blossom honeys harvested in the wider area of Hellas by professional beekeepers, were subjected to mineral content analysis using inductively coupled plasma optical emission spectrometry (ICP-OES). The main purpose of this study was to characterize the mineral profile and content of toxic metals such as lead, cadmium and chromium, and investigate whether specific minerals could assist accurately in the botanical origin discrimination with implementation of chemometrics. Twenty-five minerals were identified (Ag, Al, As, B, Ba, Be, Ca, Cd, Co, Cr, Cu, Fe, Hg, Mg, Mn, Mo, Ni, Pb, Sb, Se, Si, Ti, Tl, V, Zn) and quantified. Results showed that the mineral content varied significantly (*p* < 0.05) according to honey botanical origin, whereas lead, cadmium, and chromium contents ranged between 0.05–0.33 mg kg^−1^, <0.05 mg kg^−1^, and in the range of <0.12 to 0.39 mg kg^−1^, respectively. Fir honeys from Aitoloakarnania region showed the highest mineral content (182.13 ± 71.34 mg kg^−1^), while flower honeys from Samos Island recorded the highest silicon content (16.08 ± 2.94 mg kg^−1^). Implementation of multivariate analysis of variance (MANOVA), factor analysis (FA), linear discriminant analysis (LDA), and stepwise discriminant analysis (SDA) led to the perfect classification (100%) of these honeys according to botanical origin with the use of Al, As, Ca, Mg, Mn, Ni, Pb, Sb, Si, Zn and total mineral content. However, the higher lead content in the majority of samples than the regulated upper limit (0.10 mg kg^−1^), sets the need for further improvements of the beekeepers’ practices/strategies for honey production.

## 1. Introduction

*Apis mellifera* honeybees produce honey, a delicious natural sweetener, from nectar or honeydew secretions by adding specific substances of their own (i.e., enzymes). The basic honey components comprise sugars and water, whereas other minor components such as minerals, organic acids, enzymes, amino acids, polyphenols, fatty acids, pollen and wax originate by either bees or plants, and show diverse amounts in relation to honey botanical and geographical origin [1].

Honey may be classified into two main categories: blossom and honeydew. Typical examples of blossom honeys are thyme, citrus and heather honeys, while honeydew honeys are pine, fir, and oak honeys. However, there are numerous honey types familiar to the research community, but less known to consumers’ society. These include, for example, prairie and native vegetation honeys [2], coffee honey [3], rape, dandelion and rhododendron honeys [4], and forest and clover honeys [5].

The botanical origin of honey is officially confirmed by using the microscopic data of its pollen content (melissopalynology) [6]. In addition, the European regulation 2001/110/EC [7] sets the basic criteria for honey quality and identity. For instance, it is strictly stated that both the botanical and geographical origin of honey must be declared on the package label. Furthermore, this directive also sets specific compositional characteristics such as moisture, hydroxymethyl furfural content (HMF), free acid content, electrical conductivity, and enzymatic activity, etc. that should be taken into account.

In the literature, however, there have been numerous studies focusing on the determination of physicochemical parameters [8,9], volatile compounds [10], polyphenols [11,12], minerals [2,13,14,15,16], free amino acids [17], organic acids [17], etc., for the recognition of honey geographical and botanical origin using either official or instrumental techniques in combination with chemometrics.

Honey has a low mineral content (ca. 0.1–0.2% in blossom honeys and 1% or higher in honeydew honeys); however, mineral content analysis has gained considerable attention. Potassium has been reported to be the dominant mineral in honey, followed by other minerals such as sodium, phosphorus, magnesium, manganese, iron, copper, silicon and other trace elements [2].

There are different techniques that have been applied for the determination of honey mineral content. The most commonly used are inductively coupled plasma sector field mass spectrometry [4], inductively coupled plasma mass spectroscopy [14], inductively coupled plasma optical emission spectrometry (ICP-OES) [5,15,16], flame photometry and atomic absorption spectroscopy [2].

More recently, the European Commission Regulation [18], in an effort to guarantee honey quality and consumer safety, set the maximum level of lead to be 0.1 mg kg^−1^. However, numerous honey types are sold in the Hellenic and global market and are not mentioned, for example, in the packaging labels in regards to the levels of certain toxic metals such as lead or cadmium. In addition, there is also lack of melissopalynological analysis results. For these honeys it is usually declared in the packaging label that only the botanical and geographical origin of honey is in conformity; hence, adherent to the EU Council directive [7].

However, due to the press of the global market with products of low quality and of no specific origin, physico-chemical ‘’marker’’ analysis in combination with chemometrics may effectively assist in the determination of honey botanical and geographical origin and contribute to the establishment of health safety standards [8,9,15,19].

Based on the aforementioned, the objective of the present study was to investigate whether mineral content analysis using ICP-OES could effectively assist in the botanical origin differentiation of some lesser known Hellenic honey types such as asfaka, cotton, fir, flower, forest flowers and orange blossom honeys obtained directly from beekeepers, and provide information about lead and cadmium content of these honeys in terms of health safety standards, as relevant data for these honey types had not been reported previously.

## 2. Materials and Methods

### 2.1. Honey Samples

Due to the extent of natural limitations, nineteen honey samples (*n* = 19) of different botanical and geographical origins were collected directly from beekeepers. Flower (*n* = 3), fir (*n* = 3) and asfaka (*n* = 2) honeys were collected from Aitoloakarnania. Additional flower honey samples were collected from Samos Island (*n* = 7). The orange blossom honeys (*n* = 2) were collected from Lakonia and forest flowers honeys (*n* = 2) were collected from Zagorochoria (Ioannina, Hellas, Greece). The samples were stored in glass containers and maintained at 4 ± 1 °C until analysis. 

### 2.2. Reagents and Solutions

The chemicals used in the present study were of analytical reagent grade. All standard solutions were prepared by appropriate dilution of a multi-element standard (100 mg L^−1^) obtained from Merck (Darmstadt, Germany). The Hg standard solution (1000 mg L^−1^) was purchased from Sigma-Aldrich (Darmstadt, Germany). Nitric acid (suprapure 65%) was obtained from Merck (Darmstadt, Germany). All other solutions and dilutions were prepared with ultrapure water (Milli-Q, Millipore, Bedford, MA, USA) [15]. 

### 2.3. Preparation of Honey Samples for ICP-OES Analysis

Approximately 10 g of each honey sample were digested and mineralized by adding 65% concentrated HNO_3_ (10 mL) and by heating the mixture in a water bath at 60 °C for 30 min. The mixture was then sonicated and diluted to a final volume of 100 mL with ultrapure water before ICP-OES analysis [15]. Each analysis was carried out in triplicate (*n* = 3) and results reported are the average ± standard deviation values.

### 2.4. ICP-OES Instrumentation and Conditions

Mineral analysis was carried out using a Thermo Scientific IRIS Intrepid II XDL inductively coupled plasma-atomic emission spectrometer (Thermo Electron Corporation, Waltham, MA, USA). The emission wavelength for each element, multi-elemental analysis parameters, and method analytical characteristics are given in previous studies [15,16].

### 2.5. Chemometric Techniques

All data processing and implementation of the chemometric techniques followed were performed using the Statistical Package for the Social Sciences (SPSS) version 20.0 (IBM Corp. Armonk, NY, USA).

#### 2.5.1. Multivariate Analysis of Variance (MANOVA)

MANOVA may be characterized as a generalized form of univariate analysis of variance (ANOVA); although, unlike univariate ANOVA, MANOVA uses the covariance between outcome variables in testing the statistical significance of the mean differences [20]. It then implies a pre-evaluation step of the significance of the measured variables since it defines only the significant variables (*p* < 0.05) (considering the possible effectiveness of all variables simultaneously) assisting in the classification of samples [21].

#### 2.5.2. Factor Analysis (FA)

Factor analysis (FA) is a multivariate statistical technique used to describe the variability among correlated variables in terms of a potentially lower number of unobserved variables called factors. Factor analysis searches for such joint variations in response to unobserved, independent, and latent variables. The observed variables are modelled as linear combinations of the potential factors, plus the *error* terms. Both PCA and FA aim to reduce the dimensionality of a set of data, but the approaches followed are quite different for the two techniques. Factor analysis is clearly designed with the objective to identify certain unobservable factors from the observed variables, whereas PCA does not directly address this objective; PCA provides an approximation to the required factors [22]. There are numerous procedures designed to determine the optimal number of factors to retain in FA. In the present work, the Kaiser’s eigenvalue-greater-than-one rule (or K1 rule) was considered.

#### 2.5.3. Linear Discriminant Analysis (LDA)

LDA is a pattern recognition technique and is usually implemented after the application of MANOVA analysis or after the use of non-supervised techniques such as PCA, FA, etc. LDA uses the significant parameters defined by MANOVA (independent variables) (*p* < 0.05) to determine a linear combination of these groups of subjects which could differentiate the “a priori” known groups (grouping variables) providing exact classification rates based on the use of original and cross validation methods [16].

#### 2.5.4. Stepwise Discriminant Analysis (SDA)

In cases where a lot of predictors have arisen during the multivariate analysis, the stepwise method can be useful by automatically selecting the best variables to use in the model. We may term this procedure as an effective *haircut* of the discrimination variables for the predicting model efficacy. The stepwise method starts with a model that doesn’t include any of the predictors. At each step, the predictor with the largest F to Enter value that exceeds the entry criteria (by default, 3.84 in the SPSS program) is added to the model. The variables left out of the analysis at the last step all have F to Enter values smaller than 3.84, so no more are added. The F to Remove values are useful for describing what really happens if a variable is removed from the current model (given that the other variables remain). F to Remove value for the entering variable is the same as that of F to Enter at the previous step [23].

## 3. Results

### 3.1. Mineral Content of Asfaka, Cotton, Fir, Flower, Forest Flowers and Orange Blossom Honeys

#### 3.1.1. Abundant Minerals

The mineral content (mg kg^−1^) of the analyzed honey samples varied significantly (*p* < 0.05) according to botanical origin (Table 1). Total mineral content followed this order: Fir honey from Aitoloakarnania > forest flowers honey from Zagorochoria > cotton honey from Larissa > flower honey from Samos Island > flower honey from Aitoloakarnania > asfaka honey from Aitoloakarnania. It has been documented in the literature that honeys containing honeydew secretions or possess dark colour (i.e. honeydew, fir, forest flowers, etc.) contain a higher content of minerals compared to nectar honeys (i.e. flower, asfaka, orange blossom, thyme, etc.) [4,5,13,14,15,16]. 

In all cases, the most abundant minerals were Ca, Mg, Si, and B, followed by Mn, Fe, and Zn. 

Forest flowers honeys from Zagorochoria recorded the higher Ca content (58.30 ± 2.24, mg kg^−1^), whereas the highest Mg content (81.71 ± 1.43, mg kg^−1^), was recorded in fir honey samples from Aitoloakarnania, and may be indicative of these honey types grown in specific regions. On the contrary, Si recorded the highest content (16.08 ± 2.94, mg kg^−1^) in flower honeys from Samos Island. This is also a finding that leads to the impact of both the botanical and geographical origins of honey mineral content, given the fact that the flower honeys from Aitoloakarnania, or even the other honey types studied, did not show this trend. This observation is in agreement with the results reported by Bogdanov et al. [4].

Lesser amounts of Cu, Al, As, Ni, Pb, and Sb were recorded in all honey samples. In addition, the content of Pb, As, Cu, and Ni was below 1 mg kg^−1^ (Table 1). Only one flower honey sample from Samos Island (No.1) exceeded the value of 1 mg kg^−1^ in Cu content.

The European Commission Regulation [18] has recently set the maximum level of 0.1 mg kg^−1^ for lead in honey in an effort to ensure consumers safety and product’s quality. Lead or Cd is flourishing in the natural environment due to human activity, and honey is often subjected to contamination. Regarding Pb content, the latter exceeded this new upper limit in flower honey samples from Aitoloakarnania and Samos Island, along with those of fir honeys from Aitoloakarnania.

The content values (mg kg^−1^) obtained for Mg and Cu are within the range reported for different honey cultivars (rosemary, heather, orange blossom, and eucalyptus) cultivated in the wider area of Spain [13]. In addition, Mg and Cu content values of the present study are in agreement with the average ± standard deviation values reported for coffee honey from Brazil (82.01 ± 2.25 and 0.47 ± 0.04 mg kg^−1^, respectively).

Furthermore, Ca and Mg content values (mg kg^−1^) in asfaka honeys was significantly lower than that reported for Spanish rosemary, heather, orange blossom, and eucalyptus honeys [13]; Hellenic blossom honeys [15]; Moroccan thyme honey [16]; Egyptian clover honey [5]; or honeydew honeys from Poland [14]. On the contrary, a higher content of Ca was reported for Uruguayan and Brazilian honeys (range of 64.45 ± 25.50–77.60 ± 28.81 and 338.7 ± 14.61 mg kg^−1^, respectively) [2,3].

Furthermore, the content of Al, Mg, and Mn in the 3rd flower honey sample from Aitoloakarnania is significantly higher compared to the other flower or blossom honey samples, but within the range for fir honey samples. This is probably owed to the contribution of honeydew elements during the harvesting of flower honey in the greater area of Aitoloakarnania.

The content (mg kg^−1^) of Fe and Zn in flower honeys from Aitoloakarnania and Samos Island are in agreement with the results reported by Berriel et al. [2] involving prairie, eucalyptus and native woody vegetation honeys from different geographical zones in Uruguay. The higher Zn content was recorded for forest flowers honeys from Zagorochoria followed by orange blossom honeys from Lakonia (Table 1). The content of Zn in Swiss rape honeys (ca. 0.69 ± 0.09) is in agreement with present results involving asfaka honeys from Aitoloakarnania and flower honeys from Samos Island (0.68 ± 0.04 and 0.68 ± 0.21 mg kg^−1^, respectively).

However, the content of Ni in the present study was significantly higher in fir honeys compared to the other honey types analyzed (Table 1). Other researchers have reported lower Ni content values (range of ca. 0.03–0.06 mg kg^−1^) for Swiss acacia, chestnut, dandelion, lime, and rape honeys [4]. What is remarkable is that the Ni content values for Swiss rhododendron and mixed blossom honeys (0.15 and 0.10 mg kg^−1^, respectively) are in agreement with present results involving orange blossom honeys (Table 1). Swiss fir honeys showed a higher Ni content (ca. 1.57 mg kg^−1^) compared to present results [4].

#### 3.1.2. Minor Minerals

Some typical information about the minor mineral content data (mg kg^−1^) that are not listed in the Table 1 follow the text sequence: Barium was identified in one flower honey sample (No. 4) from Samos Island at 0.33 mg kg^−1^. In both forest flowers honey samples from Zagorochoria, Ba was recorded at 0.21 and 0.18 mg kg^−1^, respectively. Fir honey sample (No. 1) contained Ba at 0.12 mg kg^−1^. In asfaka and orange blossom honeys Ba was found at <0.06 mg kg^−1^. Finally, a cotton honey sample from Larissa contained Ba in amounts of <0.17 mg kg^−1^. These content values of Ba are in agreement with a previous work dealing with Moroccan, Spanish, Egyptian and Hellenic thyme honeys [16].

Similar content values were obtained for Cd, in which its content was <0.05 mg kg^−1^ in asfaka, cotton, forest flowers and orange blossom honeys. Cadmium was identified in an amount of <0.05 mg kg^−1^ only in 1 fir honey sample from Aitoloakarnania, whereas it was not detected in the other honey types investigated. The maximum level of 0.1 mg kg^−1^ for cadmium in honey has been suggested by the EU [18]. All honey samples analyzed conform to this upper limit and are slightly higher compared to the results reported for Swiss blossom and honeydew honeys [4]. The low Cd content values have been also confirmed in a previous work dealing with Hellenic pine, thyme, wild thyme, citrus, multifloral, and mixed citrus with erica honeys [5,15]. Chromium was identified only in 3 flower honey samples from Samos Island (Nos. 1, 5 and 7) at 0.18, 0.39 and 0.18 mg kg^−1^, respectively. In the other honey types, Cr was either not detectable (all flower honeys from Aitoloakarnania, 2 fir honeys from Aitoloakarnania (samples 1 & 2), and flower honey samples Nos. 2, 3, 4, 6 from Samos Island), or <0.12 mg kg^−1^ (asfaka, fir, cotton, forest flowers, and orange blossom honeys). These values agree with those reported for European and Mediterranean honeys [4,16].

Cobaltum content was <0.03 mg kg^−1^ in asfaka, cotton, forest flowers and orange blossom honeys, while a value of <0.11 mg kg^−1^ was recorded in a 1 fir honey sample from Aitoloakarnania. In flower honeys from Aitoloakarnania and Samos Island, cobaltum was absent. Similarly, mercury content was <0.03 mg kg^−1^ in asfaka, cotton, forest flowers and orange blossom honeys, while a value of 1.25 mg kg^−1^ was recorded in 1 fir honey sample (No. 2) from Aitoloakarnania. In flower honeys from Aitoloakarnania and Samos Island, mercury was also absent.

Molybdenum was identified in 4 flower honey samples from Samos Island (Nos. 1, 2, 3, and 6) at 0.27, 0.16, 0.21 and 0.22 mg kg^−1^, respectively, whereas in fir honey samples from Aitoloakarnania only in sample No. 2 at 0.3 mg kg^−1^. Among the rest, honey types/samples were either not detectable (all flower honeys from Aitoloakarnania, 3 flower honeys (Nos. 4, 5 and 7) from Samos Island) and 1 fir honey sample (No. 1) from Aitoloakarnania, or <0.08 mg kg^−1^.

The same holds true for Se, which was identified in higher amounts in the 5 flower honey samples from Samos Island (Nos. 1, 2, 3, 5 and 7) at 0.41, 0.42, 0.41, 0.39 and 0.64 mg kg^−1^, respectively, whereas in the rest, honey types/samples were either not detectable (all flower honeys from Aitoloakarnania, 2 flower honeys (Nos. 4, 6 from Samos Island) and 2 fir honey samples (Nos. 1 and 2 from Aitoloakarnania) or was <0.12 mg kg^−1^. In previous studies dealing with a similar topic, Se content recorded, in agreement with present results, to be higher in content in Hellenic pine and thyme honeys (0.42 ± 0.26 and 0.380.42 mg kg^−1^, respectively) [15] compared to Moroccan (0.04 ± 0.06 mg kg^−1^), Spanish (0.16 ± 0.12 mg kg^−1^) and Egyptian (0.25 ± 0.13 mg kg^−1^) thyme honeys [16].

Silver was not detected in the honey samples of the present work. Beryllium recorded a very low content (<0.06 mg kg^−1^) in asfaka, cotton, forest flowers, orange blossom honeys, whereas this amount was also identified only in 1 fir honey sample (No. 3) from Aitoloakarnania. In flower honeys from Aitoloakarnania and Samos Island it was not detected. These findings are in agreement with the results reported for Hellenic pine and thyme honeys [15].

Titanium was identified in flower honey samples (Nos. 1, 5 and 7) from Samos Island at higher amounts (0.11 mg kg^−1^), whereas in asfaka, cotton, forest flowers, and orange blossom honeys was <0.09 mg kg^−1^. Thallium was identified in low amounts (<0.02 mg kg^−1^) in asfaka, cotton, forest flowers and orange blossom honeys, in agreement with the results reported in previous studies dealing with pine, thyme, multiflower, and orange blossom honeys of the Mediterranean zone [5,16].

Finally, vanadium (V) was not identified in all flower honey samples from Aitoloakarnania and Samos Island along with 2 fir honeys from Aitoloakarnania. Amounts of V < 0.10 mg kg^−1^ were identified in the remaining asfaka, fir, cotton, forest flowers and orange blossom honeys in agreement with previous studies involving Mediterranean pine and thyme honeys [15,16].

### 3.2. Multivariate Statistics

#### 3.2.1. MANOVA

The level of significance and the ability of minerals/total mineral content to be used for the botanical origin differentiation of asfaka, cotton, fir, flower, forest flowers, and orange blossom honeys, was defined by using MANOVA. The independent variables comprised the 13 minerals and total mineral content while botanical origin was taken as the grouping (dependent) variable.

Pillai’s Trace = 4.790 (F = 8.759, *p =* 0.000 < 0.001) and Wilks’ Lambda = 0.000 (F = 44.369, *p =* 0.000 *<* 0.001) values showed the existence of a significant multivariable effect of honey minerals and total mineral content on the latter botanical origin. Ten of the 13 minerals and total mineral content were found to be significant (*p* < 0.05) for the botanical origin differentiation of honeys (Table S1).

#### 3.2.2. FA

During the FA, the K1 rule was considered in order to investigate whether the sample size could affect the reliability of analysis. The respective value of Kaiser-Meyer-Olkin (KMO) measure of sampling adequacy was 0.431 < 0.50, indicating that some minor sample issues existed. Based on this observation, the Bartlett’s test of sphericity test was also applied. Bartlett’s test of sphericity tests the hypothesis that the correlation matrix is an identical matrix, which would indicate that the variables are unrelated and therefore unsuitable for structure detection. Values less than 0.05 of the significance level indicate that a factor analysis may be useful with the set of data collected. Respective values of Bartlett’s test of sphericity were X2 = 299.314, df = 55, *p* = 0.000, indicating the effectiveness of FA in the group of samples investigated.

The extraction method was PCA. Communalities of the variables used in FA were also considered between the initial (all variables had the value 1.000) and extraction values (Table S2). It should be noted that, if the extraction values were lower than 0.3 then problems in FA analysis may arise [24]. During the analysis this problem did not arise. FA was carried by application of Varimax with Kaiser Normalization rotation method. It should be defined that the Varimax rotation of the axles is the best rotation method since it defines better the group of variables that are created and guarantees that these variables are not correlated. In addition, the possibility of co-linearity between the variables is eliminated. The structure matrix consisted of 4 principal components (eigenvalue greater than 1).

More specifically, PCA 1 (eigenvalue of 4.476) explained 40.687% of total variance and consisted of Mg, Ni, Al, Mn, and TM. PCA 2 (eigenvalue of 2.782) explained 25.291% of total variance and consisted of As, Sb, and Pb. Similarly, PCA3 (eigenvalue of 1.761) explained 16.010% of total variance and consisted of Ca and Zn. Finally, PCA 4 (eigenvalue of 1.331) explained 12.103 % of total variance and consisted of Si (Figure S1). The criterion to categorize the aforementioned variables in the four principal components was the higher absolute value of communalities (Table S3) that built the rotated component matrix. Therefore, the four principal components explained 94.091% of total variance which is a very satisfactory rate for honey botanical origin differentiation.

### 3.3. Botanical Origin Differentiation of Hellenic Asfaka, Cotton, Flower, Fir, Forest Flowers and Orange Blossom Honeys Based on Abundant Mineral Content and Linear Discriminant Analysis

Results showed that 5 discriminant functions (DFs) were formed: (i) DF1:Wilks’ Lambda = 0.000, X2 = 157.870, df = 45, *p* = 0.000, (ii) DF2: Wilks’ Lambda = 0.000, X2 = 99.277, df = 32, *p* = 0.000, (iii) DF3: Wilks’ Lambda = 0.004, X2 = 58.925, df = 21, *p* = 0.000, (iv) DF4: Wilks’ Lambda = 0.064, X2 = 28.880, df = 12, *p* = 0.000, and (v) DF5: Wilks; Lambda = 0.357, X2 = 10.810, df = 5, *p* = 0.055. It should not be forgotten, that a significant value (*p* < 0.05) of Wilks’ Lambda shows that the discriminant function is basic for the differentiation of the investigated groups. It is clear, then, that DF5 could not contribute to the botanical origin differentiation of asfaka, cotton, flower, forest flowers and orange blossom honeys.

The first discriminant function showed the highest eigenvalue (264.153) and canonical correlation of 0.998, whereas accounted for 79.4% of total variance; the second discriminant function showed an eigenvalue of 45.665, canonical correlation of 0.989 and accounted for 13.7% of total variance; the third discriminant function showed an eigenvalue of 16.486, canonical correlation of 0.971 and accounted for 5.0% of total variance; discriminant function 4 showed a lower eigenvalue (4.590), a weaker canonical correlation (0.906) and explained 1.4% of total variance. The non-significant discriminant function 5 showed the lowest eigenvalue (1.800), the weakest canonical correlation (0.802), whereas explained only 0.5% of total variance. All Dfs accounted for 100% of total variance. The unstandardized canonical discriminant functions evaluated at group means, the group centroids, had the following values: (4.289, 5.063), (−2.134, −3.331), (6.982, −1.003), (−8.418, −13.393), (10.336, 4.691), (−35.821, 5.602) for flower honey from Aitoloakarnania, Asfaka honey from Aitoloakarnania, flower honey from Samos Island, orange honey from Lakonia, fir honey from Aitoloakarnania, and forest flowers honey from Zagorochoria, respectively.

In Figure S2 it is shown that honey types are satisfactorily differentiated. The first discriminant function clearly differentiates flower honeys from Aitoloakarnania, flower honeys from Samos Island and fir honeys from Aitoloakarnania, while the second discriminant function differentiates orange blossom honeys from Lakonia and cotton honeys from Larissa. The overall correct classification rate was 100% using the original and 78.9% the cross validation method. The botanical classification rate for honey samples based on the cross validation method followed the sequence: Flower honeys from Aitoloakarnania (33.3%), asfaka honeys from Aitoloakarnania (100%), flower honeys from Samos Island (100%), orange blossom honeys from Lakonia (100%), fir honeys from Aitoloakarnania (33.3%), and cotton honeys from Larissa (100%). Regarding the low prediction rates of flower and for honeys from Aitoloakarnania, it should be stressed that from the 3 samples studied, in both cases, only 1 sample was correctly classified. In particular, of the flower honey samples from Aitoloakarnania 1 sample was correctly allocated in flower honey group, while the other two were allocated in fir honey group. Regarding fir honey from Aitoloakarnania, of the 3 samples studied, 1 sample was correctly allocated in the fir honey group, while the other two were allocated in the flower honey group from Aitoloakarnania.

### 3.4. Botanical Origin Differentiation of Hellenic Asfaka, Cotton, Flower, Fir, Forest Flowers and Orange Blossom Honeys Based on Abundant Mineral Content and Stepwise Discriminant Analysis

To investigate further, whether the correct prediction rates of honeys according to botanical origin could be improved by the selection of the most potential minerals, stepwise discriminant analysis was applied. Results showed that 5 discriminant functions (DFs) were formed: (i) DF1:Wilks’ Lambda = 0.000, X2 = 178.081, df = 35, *p* = 0.000, (ii) DF2: Wilks’ Lambda = 0.000, X2 = 92.667, df = 24, *p* = 0.000, (iii) DF3: Wilks’ Lambda = 0.012, X2 = 51.122, df = 15, *p* =0.000, (iv) DF4: Wilks’ Lambda = 0.086, X2 = 28.270, df = 8, *p* = 0.000, and (v) DF5: Wilks’ Lambda = 0.397, X2 = 10.637, df = 3, *p* = 0.014.

The first discriminant function showed the higher eigenvalue (1680,290) and canonical correlation of 1.000, whereas it accounted for 97.3% of total variance; the second discriminant function showed an eigenvalue of 36.061, canonical correlation of 0.986 and accounted for 2.1% of total variance; the third discriminant function showed an eigenvalue of 6.295, canonical correlation of 0.929 and accounted for 0.4% of total variance; discriminant function 4 showed a lower eigenvalue (3.633), a weaker canonical correlation (0.886) and explained 0.2% of total variance; discriminant function 5 showed the lowest eigenvalue (1.522), the weakest canonical correlation (0.777, whereas explained only 0.1% of total variance. All Dfs accounted for 100% of total variance. The unstandardized canonical discriminant functions evaluated at group means, the group centroids, had the following values: (15.597, 4.858), (−1.623, −0.323), (8.421, −5.520), (−20,929, −2.154), (38.207, 6.755), (−87.627, 4.378) for flower honey from Aitoloakarnania, Asfaka honey from Aitoloakarnania, flower honey from Samos Island, orange honey from Lakonia, fir honey from Aitoloakarnania, and forest flowers honey from Zagorochoria, respectively.

**Figure 1:**
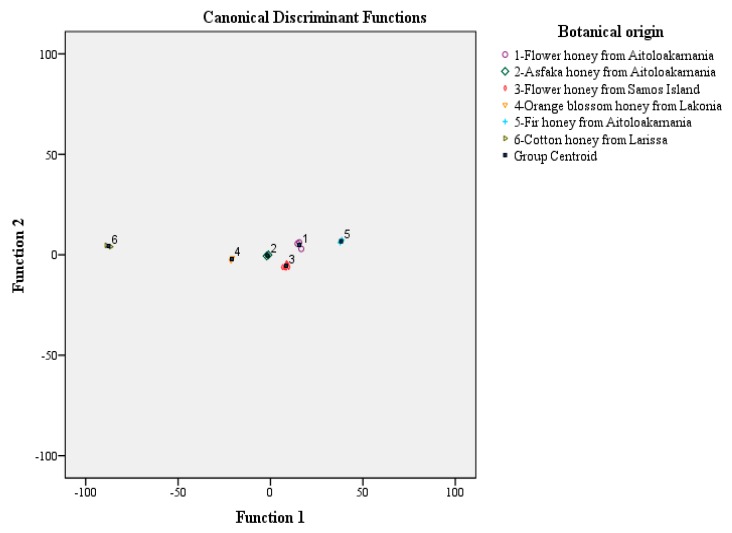
Botanical origin differentiation of Hellenic asfaka, cotton, flower, fir, forest flowers and orange blossom honeys based on abundant mineral and total mineral contents and stepwise discriminant analysis.

Stepwise discriminant analysis in Figure 1 shows that all honey types are perfectly differentiated. The first discriminant function clearly differentiates flower honeys from Aitoloakarnania, flower honeys from Samos Island and fir honeys from Aitoloakarnania, while the second discriminant function differentiates asfaka honeys from Aitoloakarnania, orange blossom honeys from Lakonia and cotton honeys from Larissa. The overall correct classification rate was 100% using the original and 100% the cross validation method. In all cases, the botanical classification rate of the honey types studied was 100%.

## 4. Overview of the Chemometrics Affinity to the Botanical Origin Differentiation of Asfaka, Cotton, Fir, Flower, Forest Flowers and Orange Blossom Honeys

The different chemometric techniques that were applied to the mineral content analysis data provided exhaustive information about their contribution to the botanical origin differentiation of asfaka, cotton, fir, flower, forest flowers and orange blossom honeys. MANOVA highlighted significant minerals (*p* < 0.05) that could contribute to the botanical origin differentiation of the aforementioned honeys. FA revealed that mineral content data could explain by a high degree (ca. 94.10%) the total variance of variables in the multi-dimensional space. Despite the minor sample size issues that existed during FA analysis by application of the KMO rule, the significant values of Bartlett’s test of sphericity showed the effectiveness of FA in the explanation of the total variance in the group of samples investigated, based on mineral content analysis data.

However, at this point some useful remarks should be recorded: According to the statistical theory, if the sample size is increased, it is reasonable to have different significant variables because the size of samples affects the confidence level [25]. In addition, it is not always a surety that by increasing the sample size, the correct classification rates of the investigated group of objects, will increase. Finally, if there were any computational problems owed to the sample size, the SPSS program would not run any analysis. Therefore, quality analysis criteria such as that of the preset study should be taken into account in cases where sample collection/sample size is, somehow, limited.

Regarding the application of LDA and SDA for the complete classification of honeys according to botanical origin based on mineral content analysis data (correct classification rates of 75% and 100%, respectively), the variables contributing the most to the overall discrimination rate were those with the highest absolute pooled-within correlation values between discriminating variables and standardized canonical discriminant functions (Table 2). These minerals may be, thus, considered as potential factors of the developed discrimination model. In that sense, for the LDA analysis these were: As (DF4), Ca (DF4), Pb (Df4), Sb (DF4), Ni (DF5), Mg (DF5), Al (DF5), Zn (DF5), and Mn (DF5). Similarly, for the SDA analysis the potential minerals were: As (DF3), Mg (DF5), Al (DF5), Si (DF5), Mn (DF5), Zn (DF5), and Ca (DF5).

Fernández-Torres et al. [13] classified eucalyptus, heather, orange blossom, and rosemary honeys from different regions in Spain by applying LDA analysis to Zn, P, B, Mn, Mg, Cu, Ca, Sr, Ba, Na, and K contents (mg kg^−1^). The overall classification rate was 97% based on the cross validation method.

Bogdanov et al. [4] classified acacia, chestnut, dandelion, lime, rape, rhododendron, fir, mountain blossom and mixed blossom honeys according to botanical origin based on Cd, Pb, Cr, Mn, Fe, Ni, Cu, and Zn content (mg kg^−1^) and LDA analysis. The overall correct classification rate was 76%. However, the individual classification rates were 100%, 100%, 100, 88%, and 83% for acacia chestnut, fir, dandelion, and rape honeys, respectively.

Chudzinska and Baralkiewicz [14], classified Polish rape, buckwheat and honeydew honeys based on K and Mn contents (mg kg^−1^) in combination with LDA analysis. These authors reported classification rates of 100%, 83% and 70% for rape, buckwheat and honeydew honeys, respectively.

Berriel et al. [2] classified prairie, eucalyptus and native woody vegetation honeys from different geographical zones in Uruguay using K, Ca, Na, Mf, Fe, Mn, Zn and Cu and discriminant analysis. The overall correct classification rates based on the original and cross validation methods were 91% and 65%, respectively.

## 5. Conclusions

Mineral content analysis implemented with different chemometric tools may accurately assist in the botanical origin differentiation of different varieties of beekeepers’ honey. The present work, apart from the mineral content characterization of asfaka, cotton, fir, flower, forest flowers and orange blossom honeys, assists in the quality control analysis of honey obtained directly from beekeepers by providing information on specific minerals, total mineral content, or the level of toxic metals such as Pb, Cd, and Cr. It is mandatory for honey consumers to know the exact quality of honey they buy, as their rights, health, and diet casualties, are well protected. Based on this ethical good, and given the fact that Pb content in most of the samples was higher than the regulated upper level, there is a great tendency for further improvement of the beekeepers’ practices for honey production.

## Figures and Tables

**Table 1:** Mineral content (mg kg^−1^) of asfaka, cotton, fir, flower, forest flowers, and orange blossom honeys.

Geographical Origin	Botanical Origin	Al	As	B	Ca	Cu	Fe	Mg	Mn	Ni	Pb	Sb	Si	Zn	Total Minerals (mg kg^−1^)
Aitoloakarnania	Flower	0.50	0.63	4.24	22.00	0.19	1.14	11.72	0.39	0.00	0.26	0.58	0.89	0.48	43.03
Aitoloakarnania	Flower	0.89	0.65	5.91	42.65	0.27	1.38	13.03	0.28	0.00	0.26	0.58	0.96	0.86	67.73
Aitoloakarnania	Flower	25.01	0.63	3.10	18.31	1.05	4.46	88.68	5.36	0.48	0.21	0.58	0.89	1.65	150.41
	Average	8.80	0.64	4.42	27.65	0.51	2.33	37.81	2.01	0.16	0.24	0.58	0.91	1.00	87.06
	±SD	14.04	0.01	1.41	13.12	0.47	1.85	44.06	2.90	0.28	0.03	0.00	0.04	0.59	56.24
Aitoloakarnania	Asfaka	1.79	0.08	3.43	11.74	0.35	0.95	14.43	0.34	0.06	0.06	0.16	4.29	0.65	38.33
Aitoloakarnania	Asfaka	2.97	0.07	3.03	13.94	0.34	1.44	14.49	0.40	0.36	0.04	0.14	4.74	0.71	42.68
	Average	2.38	0.08	3.23	12.84	0.35	1.19	14.46	0.37	0.21	0.05	0.15	4.52	0.68	40.50
	±SD	0.84	0.01	0.28	1.56	0.01	0.34	0.05	0.04	0.21	0.01	0.01	0.32	0.04	3.07
Samos Island	Flower	2.75	0.70	3.28	40.92	1.12	7.68	62.19	0.55	0.32	0.47	0.52	15.75	1.02	137.26
Samos Island	Flower	1.97	0.53	2.16	25.63	0.58	3.15	44.82	0.30	0.19	0.41	0.28	13.90	0.85	94.78
Samos Island	Flower	1.94	0.67	3.02	34.34	0.67	3.00	54.81	0.47	0.25	0.17	0.44	18.85	0.59	119.21
Samos Island	Flower	2.60	0.82	2.90	41.16	0.88	3.48	66.93	0.91	0.27	0.24	0.83	19.01	0.42	140.44
Samos Island	Flower	2.61	0.55	2.33	24.46	0.44	4.31	44.59	0.56	0.18	0.43	0.36	12.79	0.66	94.27
Samos Island	Flower	1.53	0.55	2.20	23.91	0.55	2.49	41.69	0.41	0.19	0.23	0.48	12.95	0.46	87.64
Samos Island	Flower	3.07	0.82	3.11	35.90	0.76	6.22	63.31	0.89	0.27	0.39	0.58	19.28	0.74	135.33
	Average	2.35	0.66	2.71	32.33	0.71	4.33	54.05	0.58	0.24	0.33	0.50	16.08	0.68	115.56
	±SD	0.55	0.13	0.47	7.60	0.23	1.92	10.38	0.23	0.05	0.12	0.18	2.94	0.21	22.94
Lakonia	Orange blossom	0.40	0.07	4.26	30.19	0.11	0.59	19.30	0.40	0.05	0.08	0.16	4.69	2.21	62.51
Lakonia	Orange blossom	1.33	0.06	3.83	33.85	0.10	4.76	18.40	0.47	0.20	0.06	0.13	4.50	2.21	69.88
	Average	0.87	0.07	4.04	32.02	0.11	2.68	18.85	0.43	0.13	0.07	0.14	4.59	2.21	66.19
	±SD	0.66	0.01	0.31	2.59	0.01	2.95	0.64	0.05	0.11	0.01	0.02	0.14	0.00	5.22
Aitoloakarnania	Fir	29.10	0.66	3.34	24.73	0.62	3.50	110.30	5.20	0.77	0.14	0.65	3.20	0.99	183.20
Aitoloakarnania	Fir	18.90	0.56	5.91	24.80	0.57	7.24	46.63	2.54	0.44	0.21	0.58	1.15	0.72	110.25
Aitoloakarnania	Fir	54.56	0.08	3.43	20.59	0.95	4.84	135.36	7.26	0.78	0.08	0.14	23.76	1.18	252.93
	Average	34.19	0.43	4.23	23.37	0.72	5.19	97.43	5.00	0.66	0.18	0.46	9.37	0.96	182.13
	±SD	18.37	0.31	1.46	2.41	0.21	1.90	45.74	2.37	0.20	0.05	0.28	12.50	0.23	71.34
Zagorochoria	Forest flowers	9.15	0.06	4.72	59.88	0.65	2.81	82.72	5.54	0.35	0.07	0.28	9.58	3.52	179.34
Zagorochoria	Forest flowers	8.30	0.05	4.47	56.71	0.64	2.63	80.70	5.32	0.57	0.05	0.19	8.84	3.45	171.90
	Average	8.73	0.06	4.59	58.30	0.64	2.72	81.71	5.43	0.46	0.06	0.24	9.21	3.49	175.62
	±SD	0.60	0.01	0.18	2.24	0.01	0.13	1.43	0.16	0.15	0.01	0.06	0.52	0.05	5.26
	LOD (mg kg^−1^)	0.44	0.08	0.003	0.03	0.11	0.01	0.0014	0.08	0.07	0.08	0.04	0.0011	0.0032	
	LOQ (mg kg^−1^)	1.34	0.26	0.01	0.10	0.35	003	0.0046	0.24	0.21	0.26	0.14	0.0036	0.0106	

Each sample was analyzed in triplicate (*n* = 3). LOD: limit of detection. LOQ: limit of quantification.

**Table 2:** Contribution of minerals to the discriminant function matrix during LDA and SDA.

Structure Matrix−LDA					
Minerals	Function				
	1	2	3	4	5
As	0.095	0.108	−0.186	0.476 *	−0.087
Ca	−0.083	0.053	−0.128	0.421 *	0.403
Pb	0.063	0.037	−0.243	0.397 *	−0.042
Sb	0.047	0.099	−0.050	0.275 *	−0.027
TM ^a^	0.045	0.145	0.056	0.045	0.806 *
Ni	−0.003	0.111	0.072	−0.231	0.729 *
Mg	−0.001	0.122	−0.004	−0.005	0.697 *
Al	0.020	0.122	0.201	−0.181	0.608 *
Zn	−0.215	−0.004	0.194	0.301	0.607 *
Mn	−0.037	0.164	0.156	−0.087	0.582 *
Si ^a^	0.336	0.081	0.047	−0.147	0.559 *
**Structure Matrix−SDA**					
**Minerals**	**Function**				
	**1**	**2**	**3**	**4**	**5**
Sb ^b^	0.165	0.104	0.650 *	−0.081	−0.151
As	0.033	−0.106	0.591 *	0.163	−0.203
Pb ^b^	0.294	−0.171	−0.350	0.620 *	−0.127
TM ^b^	0.020	0.105	0.307	0.016	0.816 *
Mg	0.002	0.087	0.264	−0.074	0.734 *
Al	0.015	0.188	0.043	−0.060	0.632 *
Si	0.002	−0.145	0.226	−0.139	0.600 *
Mn	−0.008	0.222	0.172	−0.067	0.598 *
Ni ^b^	0.219	0.176	0.172	0.028	0.588 *
Zn	−0.080	0.209	−0.103	0.501	0.582 *
Ca	−0.035	−0.002	0.352	0.253	0.368 *

Pooled within-groups correlations between discriminating variables and standardized canonical discriminant functions. Variables ordered by absolute size of correlation within function. * Largest absolute correlation between each variable and any discriminant function. ^a,b^ This variable was not used in the analysis.

## Supplementary Materials

The following are available online at https://www.mdpi.com/2304-8158/8/6/210/s1, Table S1: Level of significance of individual minerals and total mineral content on honey botanical origin as defined by MANOVA, Table S2: Minerals and total mineral content used to build the rotated component matrix^a^ along with communalities during extraction (Extraction method: Principal component analysis. Rotation method: Varimax with Kaiser Normalization. Rotation converged in 5 iterations. TM: total minerals. Initial communalities: all variables had the value of 1.000), Table S3: Classification ability of the SDA analysis model used for the botanical origin differentiation of asfaka, cotton, fir, flower, forest flowers, and orange blossom honeys, Figure S1: Factor analysis highlighting the principal components (structure matrix) used for the botanical origin differentiation of Hellenic asfaka, cotton, flower, fir, forest flowers and orange blossom honeys based on abundant mineral and total mineral contents. Extraction method: Principal component analysis. Rotation method: Varimax with Kaiser Normalization. Figure S1 shows only the first 3 components in dimensional space X, Y, Z. TM: total mineral content, Figure S2: Botanical origin differentiation of Hellenic asfaka, cotton, flower, fir, forest flowers and orange blossom honeys based on abundant mineral and total mineral contents and linear discriminant analysis.
