# Supplementary material for: Two-Way Characterization of Beekeepers’ Honey According to Botanical Origin on the Basis of Mineral Content Analysis Using ICP-OES Implemented with Multiple Chemometric Tools
Source: Foods. 2019 Jun 14;8(6):210. doi: 10.3390/foods8060210 (PMC6617083; doi:10.3390/foods8060210)
Supplement: Supplementary file 1 [file foods-08-00210-s001.pdf]

Supplementary Materials

Table S1. Level of significance of individual minerals and total mineral content on honey botanical origin as defined by MANOVA

Minerals	Wilks' Lambda	F	df1	df2	*p*
Al	0.306	5.899	5	13	*0.005*
As	0.180	11.858	5	13	*0.000*
Ca	0.232	8.610	5	13	*0.001*
Mg	0.391	4.048	5	13	*0.020*
Mn	0.275	6.846	5	13	*0.002*
Ni	0.350	4.821	5	13	*0.010*
Pb	0.262	7.306	5	13	*0.002*
Sb	0.414	3.684	5	13	*0.027*
Si	0.370	4.429	5	13	*0.014*
Zn	0.067	36.063	5	13	*0.000*
TM	0.336	5.148	5	13	*0.008*
B	0.464	3.003	5	13	*0.051*
Cu	0.527	2.330	5	13	*0.102*
Fe	0.594	1.780	5	13	*0.186*

F: Fisher's function; df: degrees of freedom; *p*: probability. TM: total minerals.

Table S2. Minerals and total mineral content used to build the rotated component matrix[a] along with communalities during extraction

Minerals	Component				Communalities
	1	2	3	4	
Mg	0.941	0.101	0.154	0.253	0.983
Ni	0.940				0.890
Al	0.929	-0.113	-0.254		0.941
Mn	0.927	-0.192	0.227	-0.141	0.967
TM	0.903		0.292	0.302	0.997
As		0.979	-0.104	0.106	0.982
Sb		0.956		-0.152	0.947
Pb	-0.237	0.738		0.402	0.766
Ca			0.969	0.139	0.965
Zn	0.229	-0.531	0.761	-0.186	0.948
Si	0.218			0.952	0.964

[a]Extraction method: Principal component analysis. Rotation method: Varimax with Kaiser normalization. Rotation converged in 5 iterations. TM: total minerals. Initial communalities: all variables had the value of 1.000.

Table S3. Classification ability of the SDA analysis model used for the botanical origin differentiation of asfaka, cotton, fir, flower, forest flowers, and orange blossom honeys.

Chemometric technique	Classification rate	Botanical origin	Predicted Group Membership						
			Flower honey from Aitoloakarnania (1)	Asfaka honey from Aitoloakarnania (2)	Flower honey from Samos Island (3)	Orange blossom honey from Lakonia (4)	Fir honey from Aitoloakarnania (5)	Cotton honey from Larissa (6)	Honey samples
Original[a]	Count	(1)	3	0	0	0	0	0	3
		(2)	0	2	0	0	0	0	2
		(3)	0	0	7	0	0	0	7
		(4)	0	0	0	2	0	0	2
		(5)	0	0	0	0	3	0	3
		(6)	0	0	0	0	0	2	2
	%	(1)	100.0	0	0	0	0	0	100.0
		(2)	0	100.0	.0	.0	.0	.0	100.0
		(3)	0	0	100.0	0	0	0	100.0
		(4)	0	0	0	100.0	0	0	100.0
		(5)	0	0	0	0	100.0	0	100.0
		(6)	0	0	0	0	0	100.0	100.0
Cross-validated[b,c]	Count	(1)	3	0	0	0	0	0	3
		(2)	0	2	0	0	0	0	2
		(3)	0	0	7	0	0	0	7
		(4)	0	0	0	2	0	0	2
		(5)	0	0	0	0	3	0	3
		(6)	0	0	0	0	0	2	2
	%	(1)	100.0	0	0	0	0	0	100.0
		(2)	0	100.0	0	0	0	0	100.0
		(3)	0	0	100.0	0	0	0	100.0
		(4)	0	0	0	100.0	0	0	100.0
		(5)	0	0	0	0	100.0	0	100.0
		(6)	0	0	0	0	0	100.0	100.0

[a] 100.0% of original grouped cases correctly classified. [b] Cross validation is done only for those cases in the analysis. In cross validation, each case is classified by the functions derived from all cases other than that case. [c] 100.0% of cross-validated grouped cases correctly classified.

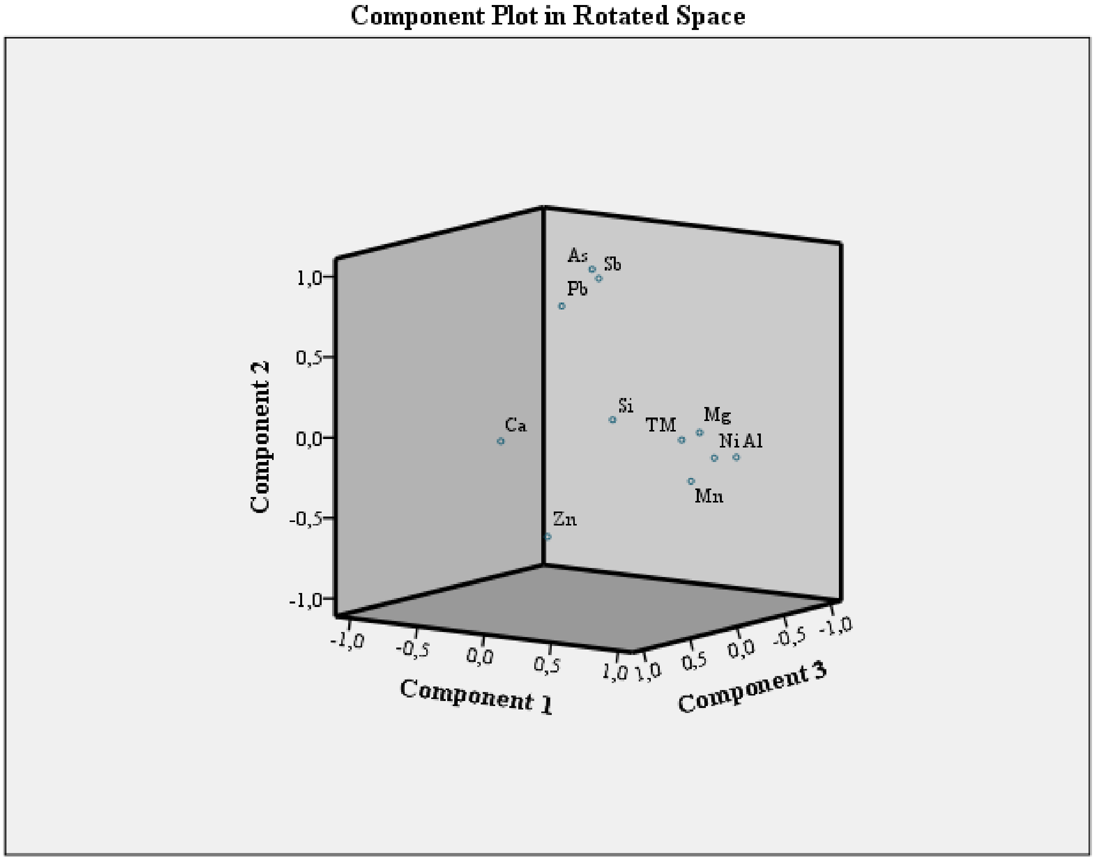

Figure S1. Factor analysis highlighting the principal components (structure matrix) used for the botanical origin differentiation of Hellenic asfaka, cotton, flower, fir, forest flowers and orange blossom honeys based on abundant mineral and total mineral contents. Extraction method: Principal component analysis. Rotation method: Varimax with Kaiser Normalization. Figure shows only the first 3 components in dimensional space X, Y, Z. TM: total mineral content.

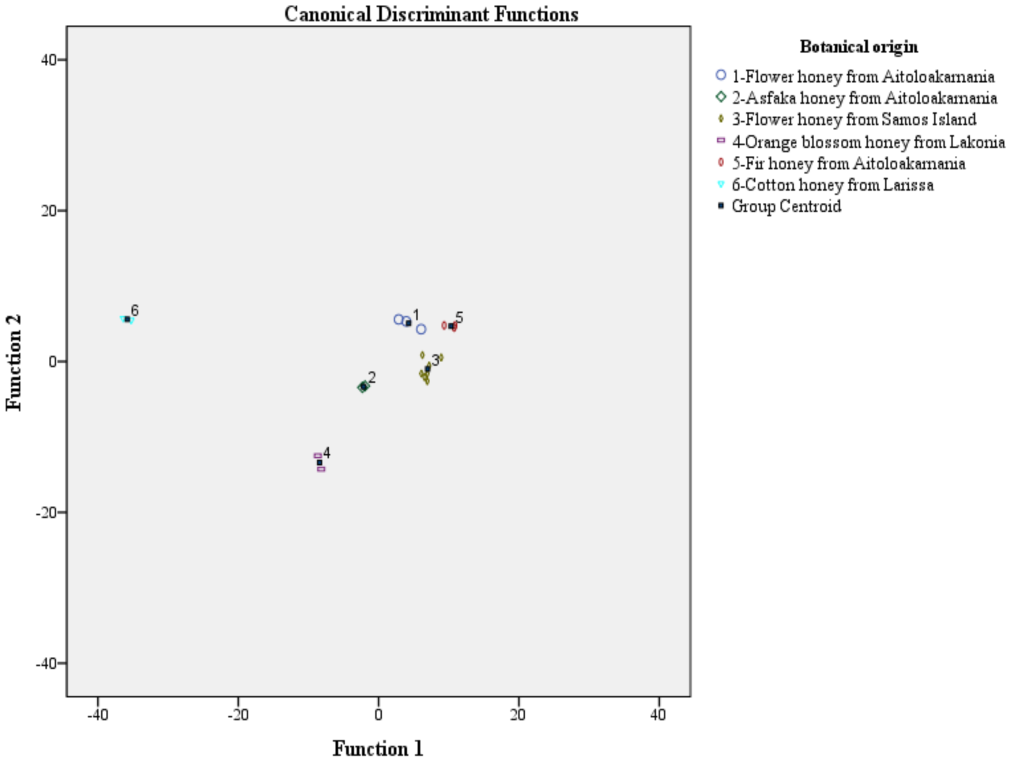

Figure S2. Botanical origin differentiation of Hellenic asfaka, cotton, flower, fir, forest flowers and orange blossom honeys based on abundant mineral and total mineral contents and linear discriminant analysis.
